# Prevalence and Correlates of Cigarette Smoking among Chinese Schizophrenia Inpatients Receiving Antipsychotic Mono-Therapy

**DOI:** 10.1371/journal.pone.0088478

**Published:** 2014-02-10

**Authors:** Yan-Min Xu, Hong-Hui Chen, Fu Li, Fang Deng, Xiao-Bo Liu, Hai-Chen Yang, Li-Guo Qi, Jin-Hong Guo, Tie-Bang Liu

**Affiliations:** 1 Department of Psychology, Normal College of Shenzhen University, Shenzhen, China; 2 Shenzhen Institute of Mental Health, Shenzhen Kangning Hospital, Shenzhen Mental Health Center, Shenzhen, China; 3 Affiliated Mental Health Center, Tongji Medical College of Huazhong University of Science and Technology, Wuhan, China; 4 Health and Family Planning Commission of Wuhan Municipality, Wuhan, China; Federal University of Rio de Janeiro, Brazil

## Abstract

**Objective:**

To investigate the prevalence rate of cigarette smoking and its socio-demographic and clinical correlates in Chinese schizophrenia inpatients receiving antipsychotic mono-therapy.

**Methods:**

This study was a cross-sectional, two-site, hospital-based survey. Four hundred and twenty-nine schizophrenia patients (male/female: 66.9% vs. 33.1%) were consecutively recruited from psychosis inpatient wards of two large specialty psychiatric hospitals in mainland China. Patients were assessed using a cigarette smoking questionnaire, the Positive and Negative Symptom Scale, the Simpson Angus Scale, the Barnes Akathisia Rating Scale, and the Abnormal Involuntary Movement Scale. Socio-demographic and other clinical data were also collected. We calculated the prevalence of current smoking in our sample as well as its indirectly standardized prevalence ratio (ISPR) using data from the 2010 Global Adult Tobacco Survey in China.

**Results:**

The prevalence rate of current smoking was 40.6% in our sample, and 57.5% in males and 6.3% in females. The ISPRs of all patients, men and women were 1.11(95%CI: 0.95∼1.29), 1.07(95%CI = 0.91∼1.24) and 4.64(95%CI = 2.12∼8.82), respectively. The overall and male-specific prevalence of current smoking did not differ significantly between patients and the general population. In multiple logistic regression analysis, male sex, older age, poor marital status, alcohol use, use of first-generation antipsychotics, longer duration of illness, more frequent hospitalizations, and more severe negative symptoms were independently associated with current smoking.

**Conclusion:**

Male Chinese inpatients with schizophrenia who received a mono-therapy of antipsychotics were not more likely to smoke than the general population. Cigarette smoking is more common in schizophrenia patients with more severe illness.

## Introduction

It has been recognized for many years that there is a much higher prevalence of smoking in schizophrenia patients than in the general population or patients with other mental disorders [1]–[3]. A meta-analysis based on worldwide studies has revealed that, the pooled smoking prevalence rates in Western schizophrenia patients were 76% for males, 50% for females, and 67% for both genders, respectively [3]. Furthermore, schizophrenia patients are not only more likely to smoke, but also smoke more heavily, have longer smoking duration, and have lower rate of smoking cessation than individuals without schizophrenia [4]. Consequently, high rate of smoking may contribute to reduction in life expectancy and excess morbidity and mortality among people with schizophrenia [5]–[8]. In addition, cigarette smoking also has been demonstrated to be associated with more frequent hospital readmissions, increased financial burden, and higher rate of attempted suicide [9]–[12].

Although cultural context contribute to cigarette smoking [13], the majority of the studies on smoking in schizophrenia patients come from Western countries, whereas few reports have come from Asian countries, especially regarding Chinese patients with schizophrenia. In recent years, a limited number of prevalence studies focusing on smoking of schizophrenia patients have been carried out in mainland China [14]–[20]. Compared with most figures reported in Western literature, the smoking prevalence rates of Chinese schizophrenia patients were relatively lower in men (median = 49%, mean = 48.7%) [15], [17], [21], and considerably lower in women (mean = 4.1%) [14], [19]. Although these Chinese studies varied with regard to sampling methods (i.e., consecutive vs. convenience sampling), samples (i.e., inpatient vs. outpatient), and definitions of current smoking (i.e., currently smoke daily vs. currently smoke every day or on some days), the notable discrepancies in the smoking rates between Chinese and Western patients may still indicate the smoking characteristics of schizophrenia patients have ethnic and/or socio-cultural differences. Thus, the findings of Western studies on smoking might not generalize to Chinese patients.

The underlying causal mechanisms of high frequency of smoking in schizophrenia patients are unclear, especially whether smoking is a risk indicator for the development of the illness, a marker of a more severe illness, or an attempt at self-medication [1], [4], [18]. Several studies viewed smoking as an environment risk factor that causes schizophrenia [22], [23]. In a historical–prospective cohort study, Weiser et al. followed 14,248 male adolescents for 4–16 years, and found a statistically significant association between the number of cigarettes smoked before the onset of illness and risk of developing later schizophrenia in smoking adolescents, with heavier smoking being associated with an almost two-fold increase in risk of schizophrenia [24]. A replication study regarding the relationship between smoking initiation and schizophrenia in Chinese population also found that daily smoking was significantly associated with vulnerability to schizophrenia [25]. On the contrary, another longitudinal study over a 27-year follow-up period revealed that smoking was associated with lower risk of subsequently developing schizophrenia and suggested that smoking might be an independent protective factor for schizophrenia [26]. In China, a study by Ma X et al. showed similar findings, in which, the authors found that smoking may delay full symptoms onset of schizophrenia by reducing some cognitive impairment in the illness and thus smoking might be a protective factor [18]. Hence, the evidence for the association between smoking and vulnerability to schizophrenia is inconclusive.

Previous studies on the clinical features of smoking schizophrenia patients also yielded inconsistent findings. Some found that smoking was significantly correlated with younger age, earlier age at onset (AAO) of illness, increased number of hospitalizations, elevated risk of suicide, higher doses of antipsychotic agents, more antipsychotic drug induced side-effects, and more severe psychotic symptoms [2], [9], [12], [18], [27]–[30]. Thus schizophrenia smokers may be more serious cases, supporting the viewpoint of smoking as a marker of more severe illness process. However, the self-medication hypothesis argues that individuals with schizophrenia smoke to alleviate negative symptoms and side effects of anti-psychotic medication [31]. Several studies found schizophrenia patients who were current smokers had significantly fewer negative symptoms [15], [17], [32] and less extrapyramidal side effects (EPS) [15], [17], [33] than non-smoking patients, supporting the self-medication theory [34]. Unfortunately, any one of these theories mentioned above, could not be used to explain all the clinical findings of existing studies, as there were still many exceptions. For example, studies found that there were no statistically significant differences between schizophrenia smokers and non-smokers with regard to the severity of negative symptoms [14], [16], [19], dystonia and dyskinesia [35], [36]. In general, research regarding the clinical characteristics of smoking individuals with schizophrenia has produced mixed results, which may be primarily due to limitations of study design. Many studies had insufficient sample sizes, used convenient samples, lacked standardized assessment tools, failed to fully address confounding (i.e., antipsychotic medication use), restricted study sample to be patients of one hospital, and, in most cases, recruited chronic rather than acute schizophrenia samples, thus only capturing data of part of the schizophrenia population.

Schizophrenia and related psychotic syndromes can be understood as disorders of adaptation to social context[37]. There is substantial evidence that schizophrenia represents the extreme of a psychosis continuum rather than a discrete illness entity, environmental factors such as taking psychoactive drugs during adolescence and young adulthood, have critical roles in the transition process from less severe levels of psychotic disorder phenotypes to schizophrenia [37]–[39]. Twin and adoption studies suggest that inherited genes make a person vulnerable to schizophrenia and then environmental factors act on this vulnerability to trigger the disorder [37], [40]. Therefore, investigating the smoking pattern of schizophrenia smokers in the context of Chinese society, especially age at regular smoking onset and heavy smoking, and the association between psychotic symptoms and smoking, might increase our understanding of the underlying mechanisms of schizophrenia. Moreover, understanding the prevalence of smoking and its clinical correlates in schizophrenia patients is important and helpful for mental health professionals to develop effective strategies to reduce its harmful consequences. Given the inconclusive findings and limitations of current studies, this study aimed to investigate the prevalence of cigarette smoking and its socio-demographic and clinical correlates in a consecutive sample of schizophrenia inpatients using a single antipsychotic agent in Wuhan and Shenzhen, China.

## Methods

### Ethics Statement

The study protocol was approved by the Ethic Committees of Wuhan Mental Health Center and Shenzhen Kangning Hospital. Before respondents were interviewed, written consent was obtained from all of the subjects and their guardians, and declarations of anonymity and confidentiality had been made. Verbal consent also obtained from each included inpatient's responsible physician. For the compensation of time spent in participating in this study, each respondent had been given a gift prior to the interview.

### Settings and subjects

This cross-sectional, two-site, hospital-based survey was conducted between January 2011 and June 2013. Subjects were recruited by consecutively screening patients with schizophrenia who attended the psychosis inpatient wards of Wuhan Mental Health Center and Shenzhen Kangning Hospital in Wuhan and Shenzhen, two of the largest cities in China. These two cities, one located in south-central area and the other located in southeastern coastal area of China, are typical examples of the metropolitan regions of mainland China, in terms of their financial conditions and levels of health care service. Both hospitals are the largest specialty psychiatric hospitals of local areas, with 610 and 450 hospital beds, respectively. They are responsible for mental health services of the residents of Wuhan and Shenzhen, and thus have a catchments area of approximately 10,020,000 and 10,547,400, respectively.

Patients who satisfied the following inclusion criteria were invited to participate in the study: 1) age 18–60 years; 2) men and women with a DSM-IV diagnosis of schizophrenia; 3) inpatients of psychosis wards; 4) had been treated with a stable dose of oral antipsychotic mono-therapy for at least four weeks before entry into the study, irrespective of the presence or absence of combination use of other non-antipsychotic drugs. Subjects with severe medical conditions, obvious intellectual disorders, active non-nicotine substance dependence, and organic psychosis, and who were prescribed two or more classes of antipsychotic agents, were excluded from this study. Although our subjects were inpatients, they were allowed to smoke regularly every day, except the time for sleep and taking drugs. Patients could easily buy cigarettes of most cigarette brands at reasonable prices in their psychiatric wards.

### Instruments and assessment

The cross-sectional data of patients' demographic and clinical characteristics and prescriptions of antipsychotic drugs were recorded using a standardized protocol and data collection procedure. Data on patients' socio-demographic variables and clinical characteristics were collected through a questionnaire designed for this study. AAO refers to age at the first psychotic symptom reported by the patients themselves or informants [41]. Alcohol user was defined as a person who drank at least one alcoholic beverage per month in the past year [42]. Case records of included subjects were additionally reviewed for the following clinical variables: number of hospitalizations, length of illness, family history of psychiatric disorders, and types and doses of current antipsychotic drugs. Unclear or inaccurate answers were confirmed again with patients themselves and/or their guardians. Discrepancies were solved by consensus between patients and their guardians or responsible psychiatrists. Because of different types of neuroleptics used, daily doses were converted into chlorpromazine equivalents (CPZ-eqs) for statistical analysis [43], [44].

We also administered a standardized cigarette smoking questionnaire to record smoking habit of each subject, including the average number of cigarettes smoked per day. Items of smoking questionnaire were adapted from the instruments of 2008 US National Health Interview Survey (hereafter refer to “NHIS definition” of smoking) [45]. It comprised of two questions. The first question, asked of all respondents, is “have you smoked at least 100 cigarettes in your entire life?” Respondents answering “yes” are classified as ever smokers, and those who answer “no” are classified as never smokers and excluded from subsequent cigarette use questions. Ever smokers are then asked a second question: “do you now smoke cigarettes every day, some days or not at all?” Respondents who answer “every day” or “some days” are classified as current smokers and those who answer “not at all” are categorized into former smokers. Heavy smokers were defined as those smoking at least 20 cigarettes daily [46].

Psychopathology of schizophrenia in the 4-week period preceding the interview was measured using the Chinese version of Positive and Negative Symptom Scale (PANSS) with subscales for positive symptoms (PANSS-P), negative symptoms (PANSS-N), and general psychopathological symptoms (PANSS-G) [47]. Drug-related extrapyramidal side effects (EPS) were assessed using Simpson Angus Scale (SAS) [48], Barnes Akathisia Rating Scale (BARS) [49], and Abnormal Involuntary Movement Scale (AIMS) [50]. The diagnostic threshold scores for the presence of pseudoparkinsonism and akathisia were ≥7 in SAS [51] and ≥2 in BARS global clinical assessment item [49], respectively. A score of 3 or more on any of the first 7 AIMS items, or a score of 2 or more on any two of the first 7 AIMS items, was taken to be indicative of dyskinesia [52]. The Chinese versions of these assessment instruments have been shown to be reliable and valid for measuring the psychotic symptoms and drug-related EPS of Chinese schizophrenia patients [53]–[56], the internal consistency of all the rating instruments (three subscales of PANSS, SAS, BARS, and AIMS) was good in this study (Cronbach α coefficients ranged between 0.74 and 0.93).

Two raters, an experienced psychiatrist and a clinical psychologist, were responsible for all of the assessments. Prior to the study, both raters underwent a training course on the use of PANSS, SAS, BARS, and AIMS. Before the beginning of this study, an inter-rater correlation coefficients ranging from 0.81 to 0.93 was obtained in 11 voluntary schizophrenia patients between the two raters for all assessment scales.

Once participants agreed to participate in this study, they were told that the main goal of this study was to help clinicians understand the relationship between schizophrenia and smoking, and the results might be helpful for the management and rehabilitation of schizophrenia. Hence, supplying real information about the smoking behaviors was of vitally importance for this study. Our raters were also required to be patient to help respondents recall their smoking details during the course of interview. The completeness of all interview and physical examination records was checked daily by the survey team leader. Errors, omissions and other flaws were solved during the fieldwork period of this survey.

### Statistical analysis

For the analysis, we used the SPSS software for Windows, version 15.0 (SPSS Ltd.). According to the smoking status diagnosed by NHIS definition, prevalence rates for ever, current and former cigarette smoking were calculated. Because the number of former smoker in our sample was very small (N = 3), the following univariate and multivariate analyses only focused on current and never smokers. In univariate analysis, the comparison between current smokers and never smokers with respect to socio-demographic and clinical characteristics was performed by independent sample t-test, Mann-Whitney U test, and chi-square test as appropriate. Multivariate logistic regression was used to identify factors associated with current smoking. Associations between current smoking and all potential predictors were assessed in univariate analyses, followed by multivariate analyses of the selected subsets. A variable was selected if it was statistically significant at the nominal two-sided 0.05 level in univariate analysis. We quantified associations of predictors and current smoking by calculating odds ratios (ORs) with 95% confidence intervals (CIs) for each variable.

To clarify whether schizophrenia inpatients have higher current smoking rate than Chinese general population, we introduced the latest smoking data from the 2010 Global Adult Tobacco Survey in China (2010 GATS China) [57]–[59] as the comparison reference. The 2010 GATS China was a nationally representative household survey of non-institutional men and women aged 15 and older, and was conducted between December 2009 and March 2010. The survey was completed with high response rate (96.0%) and high qualities by strict quality control measures, and successfully collected 13,354 Chinese adults' tobacco use data. Since our study sample and the external standard population, 2010 GATS China sample, were not comparable in terms of age and gender structures, we compared the smoking rates between schizophrenia and the general population by calculating the Indirectly Standardized Prevalence Ratios (ISPRs) [60], [61]. Specifically, this indirect standardization approach applied the stratum specific smoking rates (i.e., age and gender specific rates) of 2010 GATS China population to the number of individuals in the corresponding stratum in our sample. An expected number of smokers was generated for each stratum, and the total expected was used in the denominator. The numerator was the total number of observed smokers in our schizophrenia sample. ISPR was the ratio of the observed number of smokers in our study sample over the expected number of smokers in the general population. Its 95%CI was estimated through formulas proposed by Rothman and Boice [62].

## Results

### Characteristics of subjects

Out of 976 schizophrenia inpatients who were screened during the study period, 617 were on antipsychotic mono-therapy treatment and fulfilled the inclusion criteria. Among them, 56 refused, 93 did not complete assessment, and 39 were ineligible for other reasons, leaving 429 (69.5%) patients completed the interview and physical examination. The mean age at the time of this survey was 33.1 years (standard deviation [SD] = 10.2, range = 18∼60), and 66.9% of the subjects were male. The numbers of patients using promethazine, perphenazine, chlorpromazine, sulpiride, paliperidone, clozapine, risperidone, quetiapine, olanzapine, amisulpride, and aripiprazole were 2, 19, 76, 5, 1, 117, 95, 24, 53, 2, and 35, respectively. Therefore, for the antipsychotics used, the second-generation antipsychotic (SGA) mono-therapy was used more often compared to the first-generation antipsychotic (FGA) (77.4% vs. 22.6%) mono-therapy. Other socio-demographic and clinical characteristics of the schizophrenia inpatients are displayed in the first three columns of Table 1.

**Table 1:** Socio-demographic and clinical characteristics of inpatients with schizophrenia, split by smoking status.

	All subjects (N = 429)		Current smokers (N = 174)		Never smokers (N = 252)		Statistics	
Variables	n	%	n	%	n	%	?2	P
Study site (Wuhan)	306	71.3	119	68.4	187	74.2	1.71	0.19
Male	287	66.9	165	94.8	119	47.2	105	<0.001
Without medical insurance	232	54.1	98	56.3	133	52.8	0.52	0.47
Marital status							16.74	<0.001
Married/re-married/co-habiting	146	34	43	24.7	101	40.1		
Never-married	227	52.9	97	55.7	129	51.2		
Divorced/widowed/separated	56	13.1	34	19.5	22	8.7		
Being unemployed	178	41.5	73	42	104	41.3	0.02	0.89
Alcohol use	121	28.2	82	47.1	37	14.7	53.8	<0.001
Family history of psychiatric disease	92	21.4	37	21.3	53	21	0.003	0.95
Taking FGAs	97	22.6	60	34.5	37	14.7	22.9	<0.001
Pseudoparkinsonism	60	14	6	3.4	54	27.5	60.3	<0.001
Akathisia	94	21.9	21	12.1	73	29	17.1	<0.001
Dyskinesia	85	19.8	31	17.8	54	21.4	0.84	0.36
	Mean	SD	Mean	SD	Mean	SD	t/Z	P
Age (years)	33.1	10.2	35.5	9.7	31.3	10.2	4.19	<0.001
Education level (years)	11.2	3.4	11.1	2.8	11.4	3.8	1.01	0.32
Age at onset (years)	24.9	7.4	25.7	7.8	24.2	7.1	2.11	0.035
Length of illness (years)	7.6	8.7	8.9	8.4	6.7	8.9	3.44	0.001*
Duration of antipsychotic treatment	6.2	7.9	7.7	8.2	5.1	7.6	4.46	<0.001*
Number of hospitalizations	3.3	3.7	4.4	4.7	2.6	2.5	5.48	<0.001*
Neuroleptic dose (CPZ eqs, mg/day)	372.6	318.7	390.8	312.3	353.5	333.1	0.67	0.45*
PANSS total score	56.7	21.4	61.5	24	52	17.1	4.77	<0.001
PANSS-P subscore	16.2	6.8	17.3	7.4	15.1	5.8	3.43	<0.001
PANSS-N subscore	13.2	6.1	14.2	6.6	12.2	5.3	3.46	<0.001
PANSS-G subscore	27.3	12.8	30	14	24.7	10.8	4.4	<0.001

Note: FGA, first-generation antipsychotics. CPZ eqs, chlorpromazine equivalents; PANSS, positive and negative symptom scale; *Mann-Whitney U test.

### Smoking patterns of schizophrenia inpatients

Of the 429 patients, 177 (41.3%) were identified as ever smokers, 3 as former smokers (0.7%) and 174 as current smokers (40.6%); 57.5% of male (N = 165) and 6.3% of female patients (N = 9) were current smokers. Among the current smokers, the mean number of cigarettes consumed per day was 16.1 (SD = 9.0, range = 2–40), while 15.2% of them smoked ≤ 9 cigarettes daily, 43.5% of them smoked 10–19 cigarettes, and 41.4% smoked ≥ 20 cigarettes (heavy smoking). For current smokers, the mean age at regular smoking onset was 20.6 (SD = 4.6) years, and the mean AAO for schizophrenia was 24.9 (SD = 7.4) years, with 75.8% starting regular smoking before the onset of schizophrenia. The age of regular smoking start in patients was significantly earlier than their age of illness onset (paired-samples t test, t = 13.38, P<0.001).

The ISPRs of all patients, male and female patients were 1.11(95%CI: 0.95∼1.29), 1.07 (95%CI: 0.91∼1.24) and 4.64(95%CI: 2.12∼8.82), respectively (Table 2). These results mean that only female patients had a statistically higher prevalence of current smoking than the general population, whereas all patients and female patients did not. Compared with smokers of the general population, current smokers with schizophrenia had significantly lower percentage of heavy smokers (41.4% vs. 51.9%, Z = 2.77, P = 0.023). There was no statistically significant difference in age of initiation of regular smoking between current smoking patients and the general population (mean age at smoking initiation in 2010 GATS China  = 21.2 years, one-sample t test, t = 1.72, P = 0.087).

**Table 2:** Indirectly standardized prevalence ratios of current smoking in schizophrenia inpatients, calculating using the results of 2010 Global Adult Survey in China as the standard populations.

		Age group (years)						
Sample	Parameter for indirect standardization	16∼24	25∼34	35∼44	45∼54	55∼60	Total	Indirectly standardized prevalence ratio (95% CI)
Males	No. of subjects	59	105	67	48	8	287	1.07(0.91, 1.24)
	Prevalence of current smoking in patients (%)	33.9	64.8	58.2	70.8	50	57.5	
	Age-specific prevalence of current smoking in general population (%)	33.6	53.0	63.7	66.3	58.9	—	
	No. of observed current smokers	20	68	39	34	4	165	
	No. of expected current smokers	19.8	55.7	42.7	31.8	4.7	154.7	
Females	No. of subjects	49	41	31	20	1	142	4.64(2.12, 8.82)
	Prevalence of current smoking in patients (%)	8.2	2.4	6.5	10	0.0	6.3	
	Age-specific prevalence of current smoking in general population (%)	0.7	0.3	2.4	3.5	2.8	—	
	No. of observed current smokers	4	1	2	2	0	9	
	No. of expected current smokers	0.34	0.12	0.74	0.70	0.03	1.94	

### Correlates of current smoking

The socio-demographic and clinical characteristics of current and never smokers are shown in the fourth to seventh columns of Table 1. Univariate analysis (the last two columns of Table 1) showed that current smokers were more likely to be male, be in poor marital status (never married, divorced, widowed, or separated), use alcohol, take FGAs, have less pseudoparkinsonism, have less akathisia, be older, have later AAO, have longer duration of illness, receive long-term antipsychotic treatment, be hospitalized more frequently, be scored higher in PANSS, PANSS-P, PANSS-N, and PANSS-G scales. Multivariate logistic regression analysis (Table 3) considering all variables statistically significant in the univariate analysis (with a Backward: Wald χ2 selection method) revealed that male sex, older age, poor marital status, alcohol use, use of FGAs, longer duration of illness, more frequent hospitalizations, and more severe negative symptoms remained independently associated with smoking after adjustment for all other variables.

**Table 3:** Multiple logistic regression for correlates of current smoking.

Variables	Reference category	Beta	Standard error	Wald χ2	P	OR(95%CI)
Male gender	Female gender	2.90	0.41	51.23	<0.001	18.18(8.20, 40.0)
Age (years)	—*	0.09	0.02	17.42	<0.001	1.09(1.05, 1.14)
Never-married	Married/re-married/co-habiting	1.16	0.35	10.76	0.001	3.18(1.59, 6.34)
Divorced/widowed/separated	Married/re-married/co-habiting	1.43	0.45	9.90	0.002	4.17(1.71, 10.14)
Alcohol use	No alcohol use	1.87	0.31	35.73	<0.001	6.50(3.52, 12.00)
FGAs	SGAs	1.38	0.33	17.52	<0.001	3.98(2.08, 7.58)
Length of illness (years)	—*	0.09	0.02	16.15	<0.001	1.09(1.05, 1.14)
Number of hospitalizations	—*	0.16	0.05	9.92	0.002	1.17(1.06, 1.29)
PANSS-N subscore	—*	0.06	0.03	4.54	0.013	1.06(1.01, 1.12)

Note: *continuous variable.

## Discussion

This study demonstrated that the prevalence of current smoking was 40.6% and the ISPR was 1.11 in Chinese schizophrenia inpatients currently receiving a mono-therapy of antipsychotics. However, both the overall and male-specific prevalence rates of current smoking did not differ significantly between patients and the general population. Only female Chinese inpatients with schizophrenia seem to have higher smoking risk than the general population, but the absolute difference (equals to 3.9%) between the two prevalence rates is really too small. Considering the finding that the female-specific prevalence (6.3%) was too low, and the comparable prevalence between the whole sample and the general population, it is difficult to conclude that Chinese inpatients with schizophrenia are more likely to smoke than the general population. This study further demonstrated that current smokers of patients had significantly lower rate of heavy smoking than those of the general population, indicating that Chinese schizophrenia smokers are not heavy cigarette smokers. Although we found the majority of smokers (75.8%) started regular smoking before the disease onset, the mean age of starting to smoke (20.6 years) was similar to that of the general population; these phenomena might not support the viewpoint that schizophrenia patients start regular smoking in greater numbers during adolescence than the normal population in Western countries [63]. Furthermore, the similar overall prevalence of smoking between our patient sample and the general population may indicate that smoking in Chinese schizophrenia patients is an ingrained habit. In Chinese culture, smoking is a deeply rooted tradition, as most of the Chinese people have already been accustomed to offering others cigarettes or smoking cigarettes provided by others as a gesture of hospitality and friendship[64]. The studied psychiatric hospitals also supplied relatively free environments to let their patients smoke. There is a possibility that Chinese schizophrenia patients and the general population had comparable prevalence of smoking before hospitalization. Under the circumstances, individuals with schizophrenia would still keep their smoking habit even after their admission in the hospital, therefore, no statistically significant difference in the overall prevalence of current smoking was found between the two groups in this study. Another finding that almost all of the ever smokers were current smokers in our study sample seems consistent with such hypothesis.

In addition to showing the differences in smoking characteristics between individuals with schizophrenia and the general population, our study also found lower current smoking prevalence, including gender-specific and overall prevalence, of schizophrenia inpatients than those reported previously in Western studies [US (males: 91%, females:70%) [65], Canada (both genders: 63%) [66], Germany (males: 70%) [67], Spain (males: 81%, females: 13%) [68], and Ireland (males: 92% females: 82%) [69]]. These low prevalence rates in our study are somewhat consistent with the findings of previously published Chinese schizophrenia inpatient studies (males: 42.2%∼52.0%, females: 4.5%∼6.3%) [14], [18], [20]. The discrepancies in the smoking rates between Chinese and Western studies could be due to the exclusion of patients with alcohol/heroin dependence (smoking rate in such patients is expected to be high) [70], and the potential selection bias that only patients using a single antipsychotic agent was included in this study. Moreover, in most literature from western countries, the smoking rate of female schizophrenia patients is very close to that of males[3], but in Chinese schizophrenia patients, women's smoking prevalence is obviously lower than men's. The high ratio of male-to-female smoking prevalence rates is in line with that of the normal population in 2010 GATS China survey[57]. The dramatic gender difference reflects the cultural differences between China and western countries. In general, China is still a male dominated society, and smoking is regarded as the patent and privileges of men. Chinese females who smoke are typically viewed as women with either a wild streak or loose morals. Thus the cultural resistance against women smoking in modern China remains very strong[16], this cultural phenomenon should also be applicable to Chinese patients with schizophrenia, which leads to the low smoking rate in female patients compared with western female patients.

When we study the association between smoking and schizophrenia, as measured by OR, it is noteworthy that the estimated ORs have close relationship with the smoking prevalence rates of the general population, because the conclusions regarding whether patients have higher, lower or comparable risk of smoking, highly depending on the control population used. For example, two studies conducted in Turkey and US reported similar smoking rates in their schizophrenia sample (50% and 51%) [71], [72], but the corresponding smoking rates in Turkey and US general population were quite different (43% and 23%), therefore, a non-significant OR of 1.3 for Turkey patients and a statistically significant OR of 3.5 for US patients were obtained [3]. In our study, we not only found a lower prevalence of smoking, but also revealed a comparable smoking prevalence between patients and the general population. Schizophrenia smokers in our sample are less likely to be heavy smokers as Western smokers with schizophrenia [2]. As a result, smoking might not be associated with the vulnerability to schizophrenia in Chinese population. The potential socio-cultural, biological and public anti-smoking policy factors for these differences need to be further explored.

Analyses of correlates of current smoking revealed that, in schizophrenia inpatients, smoking was more prevalent among patients who were male, were older, were in poor marital status, used alcohol, took FGAs, had longer illness duration, had multiple hospitalizations, and had more severe negative symptoms. These findings indicate that the potential etiologies of smoking in schizophrenia are complex and multifactorial. In this study, male sex, older age (below 60 years old), and unmarried status were identified as significantly risk factors for smoking, these findings are in line with previous reports for both the general population [59], [73] and schizophrenia patients [16], [19], [20]. In general, alcohol drinkers usually smoke and vice versa, as both behaviors are strongly influenced by some shared genetic factors [74]. In agreement with the findings of earlier studies [16], [17], smoking was significantly associated with alcohol use in our schizophrenia sample. Several studies have shown a higher prevalence of smoking in schizophrenia patients with lower educational attainment [2], [19]; however, we did not find such association in our sample, which is consistent with three previous studies [12], [16], [17]. This discrepancy might be attributable to the differences in sample characteristic. Also, other risk factors could mask or affect the role of education level.

It has been suggested that individuals with schizophrenia use tobacco to gain relief from EPS induced by antipsychotic medication. Cigarette smoking increases the activity of hepatic CYP 1A2 enzymes, thus decreasing the concentration of antipsychotic drugs [28], [75]. Therefore, patients on FGAs are more likely to smoke as a result of much EPS from FGA treatment. This study partly replicates earlier findings [15], [16], [31], [33], as its univariate analysis found that a higher likelihood of smoking was associated with less EPS (pseudoparkinsonism and akathisia) and use of FGAs. Smoking could increase clearance of antipsychotics, which in turn makes schizophrenia smokers require higher dosages of antipsychotics than non-smokers, but there was no statistically significant difference in the neuroleptic dosage between smokers and never-smokers in the present study. Moreover, more severe negative symptoms were found to be correlated with current smoking. These findings might suggest the undertreatment of inpatients with schizophrenia. Another reasonable explanation for this phenomenon is that there was a significantly higher proportion of FGA use in smokers, but FGAs are not good at reducing the negative symptoms. Hence, the association between more severe negative symptoms and smoking was found. Although nicotine might alleviate negative symptoms of smokers through stimulating release of dopamine and serotonin in the brain [76], these positive effects might have been weakened by the increased clearance of antipsychotics and undertreatment. Other factor correlated with EPS may mask the role of EPS to smoking, so the statistically significant association between less EPS and smoking disappeared in the multiple logistic regression model. The finding of more severe negative symptoms in smokers when compared to never-smokers is similar to the results of several previous studies [29], [30], [77].

After adjusting confounders, smoking was still associated with more hospital admissions and longer illness duration; both findings indicated that our smoking cases have a more severe disease course. Previous studies also support these findings [15], [17], [29], [30], [77]. Presence of smoking has been reported to be associated with increased risk of hospital readmission and longer duration of hospitalization [78]. The increased severity of illness also may be attributed to high prevalence of alcohol use, as alcohol consumption was founded to be associated with increased risk of hospital admissions in cases with physical disease [79]. There is evidence that severe, treatment resistant schizophrenia is associated with smoking in patients with schizophrenia regardless of gender or alcohol use [80]; this is similar to our finding that smoking was related to more severe illness.

The strength of this study was ensuring the analysis on the association of smoking with types and doses of antipsychotic agents in subjects receiving neuroleptic mono-therapy treatment, by eliminating the masked effect of patients receiving multiple antipsychotic medications. However, this study has several limitations that should be noted. First, our study sample consists of inpatients on antipsychotic mono-therapy only and can’t represent all patients with schizophrenia. Therefore, the results may not be applicable to outpatients and inpatients on antipsychotic poly-therapy. In addition, our sample was selected from specialty psychiatric hospitals of two large cities in mainland China. Although the prevalence and correlates identified in our study seem consistent with several findings in previous reports, whether they also apply to other institutions of China is still questionable. So the findings from this study should be generalized with caution. Second, our study was a cross-sectional, observational study in nature, thus the cause-consequence relationships between smoking and risk factors could not be created. Third, the definition of smoking used in the present study is broader, because it defines a current smoker as an ever smoker who currently smokes daily or on some days [81]. On the contrary, most of the previous clinical studies restricted current smokers to be ever smokers who currently smoke daily. Hence, we may overestimate the smoking prevalence of our schizophrenia sample. Fourth, this study used a questionnaire to record the smoking status of schizophrenia patients, which is not as accurate as several laboratory indicators, such as measuring the level of cotinine (a metabolite of nicotine) in the blood samples. Although we had taken some quality control measures to minimum the measurement bias, biases due to memory effect and social desirability response might still be inevitable in the present study. Finally, since the number of identified female smokers was very limited, males accounted for 95% the smokers; on the other hand, the sample size of female patients in our sample is also small. Thus, those identified correlates of smoking may not be applied to female schizophrenia patients and the prevalence estimation of smoking in female patients is unstable. The smoking characteristics of female people with schizophrenia need to be further studied.

## Conclusions

In summary, smoking in Chinese male schizophrenia inpatients, who were receiving antipsychotic mono-therapy, is as prevalent as the general population, and the overall prevalence of smoking in Chinese schizophrenia inpatients is lower than most figures reported in Western literature. Smoking had relationship with gender, age, and marital status; Illness severity-related indicators seemed to have close relationship with smoking in this patient population. Smoking may serve as an important clinical marker of severe illness in individuals with schizophrenia. Given the harmful consequences and its association with more severe illness, effective measures to promote smoking cessation in schizophrenia should be implemented in Chinese patients with schizophrenia.
